# SIRT1: potential target in glucocorticoid-resistant diseases

**DOI:** 10.3389/fimmu.2025.1514745

**Published:** 2025-05-09

**Authors:** Jun Xie, Siyi Che, Jiao Liu, Xiaoru Long

**Affiliations:** Department of Respiratory Medicine, Children’s Hospital of Chongqing Medical University, National Clinical Research Center for Child Health and Disorders; Ministry of Education Key Laboratory of Child Development and Disorders, China International Science and Technology Cooperation Base of Child Development and Critical Disorders, Chongqing Key Laboratory of Child Rare Diseases in Infection and Immunity, Chongqing, China

**Keywords:** SIRT1, glucocorticoid resistance, glucocorticoid receptor, acetylation, T cell

## Abstract

Glucocorticoid resistance is a challenging problem in clinical practice. Increasing glucocorticoid sensitivity and reducing resistance are important in the management of certain diseases. In steroid-resistant airway inflammatory diseases, glucocorticoid receptor (GR) expression is reduced, and impaired GR nuclear translocation is closely related to glucocorticoid resistance. Histone deacetylase SIRT1 regulates steroid hormone receptor activity and interacts with the androgen receptor and GR. In some glucocorticoid-resistant diseases, SIRT1 expression is reduced. Here, we review recent advances in the role of SIRT1 in regulating glucocorticoid signaling. First, we describe the structure, tissue expression, and subcellular localization of SIRT1. We also discuss the molecular mechanisms by which SIRT1 regulates glucocorticoid activity and its association with GR, as well as the mechanisms and roles of SIRT1 in several common glucocorticoid-resistant diseases. SIRT1 may serve as a potential therapeutic target, providing an opportunity for the treatment of glucocorticoid-resistant diseases.

## 1 Introduction

Glucocorticoids are steroid hormones used to treat inflammatory and autoimmune diseases. However, with their widespread use, the occurrence of glucocorticoid insensitivity and resistance has also increased. Several diseases, including asthma, nephrotic syndrome, and allergic rhinitis, have been shown to develop glucocorticoid resistance (1–3). Glucocorticoids need to bind to the cytoplasmic glucocorticoid receptor (GR) to enter the nucleus and exert anti-inflammatory effects through transcriptional activation or inhibition. Under normal physiological conditions, the distribution of GR in the cytoplasm and nucleus is in a state of dynamic balance. When hormone secretion increases or exogenous hormone therapy is given, GR and glucocorticoids immediately accumulate in the nucleus. In steroid-resistant airway inflammatory diseases, the expression of GR is reduced, thus limiting its ability to enter the nucleus, which is closely related to glucocorticoid resistance (4, 5).

Histone deacetylases (HDACs) and their inhibitors play critical roles in glucocorticoid-resistant diseases (6, 7). Reduced HDAC2 activity is associated with the modulation of glucocorticoid insensitivity in diseases such as asthma (8). HDAC family member sirtuin 1 (SIRT1) is reported to play a critical role in the regulation of glucocorticoid signaling (9, 10). The human SIRT family includes seven members (SIRT1–SIRT7) that participate in many physiological and pathological processes, including cellular energy metabolism, DNA repair, oxidative stress, and inflammatory response by deacetylating a series of important proteins (11–13). However, the role of SIRT1 in glucocorticoid-resistant diseases is unclear. Hence, in this paper, we summarize the latest advances in the role of SIRT1 in regulating glucocorticoid activity and glucocorticoid-resistant diseases.

## 2 SIRT1 gene and structure

**Figure 1:**
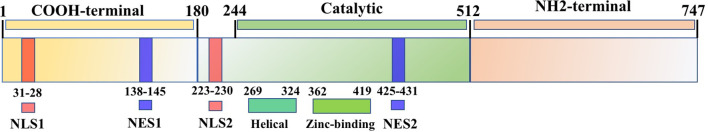
SIRT1 protein structure. SIRT1 protein contains 747 amino acid residues, consisting of NH2-terminal, catalytic, and COOH-terminal domains, as well as two nuclear localization signals (NLS) and two nuclear export signals (NES).

It is widely acknowledged that the silencing information regulator (SIR) complex confers longevity in yeast. Among the seven types of SIRT found in mammals, SIRT1 is located at chromosome 10q21.3 and consists of eight introns and 11 exons, with a length of 33 715 bp. The human SIRT1 protein contains 747 amino acid residues, consisting of NH2-terminal (513–747 residues), catalytic (244–512 residues), and COOH-terminal domains (1–180 residues) (14). The catalytic core consists of two domains: i.e., a highly conserved NAD^+^-binding domain and a helical (269–324 residues) and zinc-binding domain (362–419 residues). Catalytic reactions are initiated by the binding of acetylated targets and NAD+ (15). The SIRT1 protein contains two nuclear localization signals at residues 31–38 and 223–230 and two nuclear expert signals at residues 138–145 and 425–431 (16) (**Figure 1**). Nuclear import and export sequences are considered to be the regulatory mechanism underlying the nucleocytoplasmic shuttling of SIRT1. The 2–268 region interacts with histones 1–4 (17) and the circadian locomotor output cycles kaput (CLOCK) protein (18), while residues 256–259 are required for interactions with cell cycle and apoptosis regulator 2 (CCAR2) (19).

## 3 SIRT1 expression and localization

**Figure 2:**
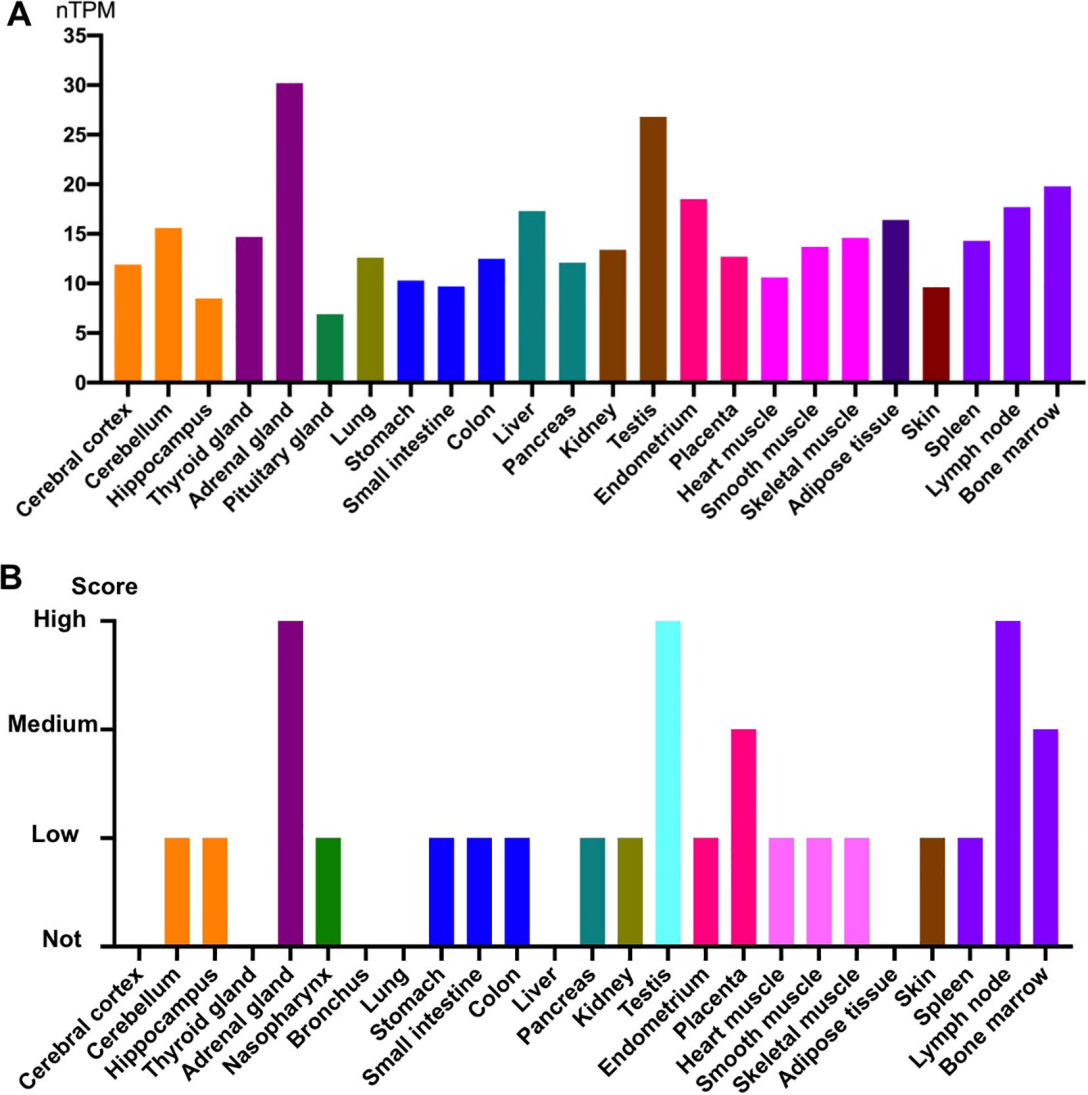
SIRT1 gene and protein expression in different human tissues. **(A)** Normalized expression (nTPM) levels of SIRT1 in different tissues. SIRT1 showed high expression in the adrenal glands and testis. **(B)** Protein expression of SIRT1 in different tissues. Protein expression was high in the adrenal gland, testis, and lymph node. All data were obtained from the Human Protein Atlas.

SIRT1 is expressed in all tissues, particularly in testis and endocrine tissues (20). **Figure 2** shows the protein and RNA expression levels in different human tissues (from the Human Protein Atlas). While SIRT1 is localized in the nucleus of HeLa cells and HEK293 cells (21, 22), it is also found in the cytoplasm of A549 and human bronchial epithelial cells (23, 24). Furthermore, the subcellular localization of SIRT1 can change under specific circumstances. In BEAS-2B cells, cigarette smoke extract can induce nuclear translocation of SIRT1 from the cytoplasm and is associated with strong induction of several antioxidant genes (24). In murine microglial cells, cobalt chloride treatment can prevent SIRT1 nuclear localization, leading to neuronal damage (25). As SIRT1 deacetylates histones and various nonhistone proteins, aberrant changes in its subcellular localization may affect its function.

## 4 SIRT1 interacts with glucocrticoid signaling

### 4.1 Glucocorticoid induce SIRT1 expression

SIRT1 regulates the activity of glucocorticoids, while glucocorticoids also influence the expression of SIRT1. Dexamethasone reduces SIRT1 expression and enzymatic activity by inducing miR-128, which is known to directly target SIRT1 in pig preadipocytes (26). Furthermore, dexamethasone-induced expression of miR-34a can suppress SIRT1 deacetylase activity, led to decreased dexamethasone-induced cell death responses in malignant multiple myeloma cells (27). In mesenchymal stem cells, dexamethasone treatment for 24 h reduced SIRT1 expression (28). Moreover, in rats with adjuvant-induced arthritis, glucocorticoid treatment reduced the increase in SIRT1 expression and accompanying inflammation in PBMCs and liver (29). These studies indicate that glucocorticoid treatment down-regulates SIRT1 expression, which could further impair glucocorticoid activity in certain diseases.

### 4.2 SIRT1 regulates glucocorticoid activity

Growing evidence suggests that SIRT1 regulates steroid hormone receptor activity. SIRT1 generally inhibits androgen receptor (AR), estrogen receptor (ER), and mineralocorticoid receptor (MR) activity (30), but has different effects on glucocorticoid signaling activity. Prednisolone is reported to inhibit adriamycin-induced vascular smooth muscle cell senescence and inflammation through the SIRT1-AMP-activated protein kinase(AMPK)signaling pathway (31), with down-regulation of SIRT1 augmenting the effects of prednisolone on inflammation and senescence, and up-regulation of SIRT1 attenuating the effects on cellular senescence. Furthermore, SIRT1 promotes glucocorticoid induced anti-inflammatory activity but inhibits uncoupling protein-3 (UPC3) gene transcription, a mitochondrial membrane transporter induced by glucocorticoid that protected skeletal muscle cells from oxidative stress damage (32). Thus, these studies suggest that SIRT1 exerts variable functions on glucocorticoid signaling under different conditions.

**Figure 3:**
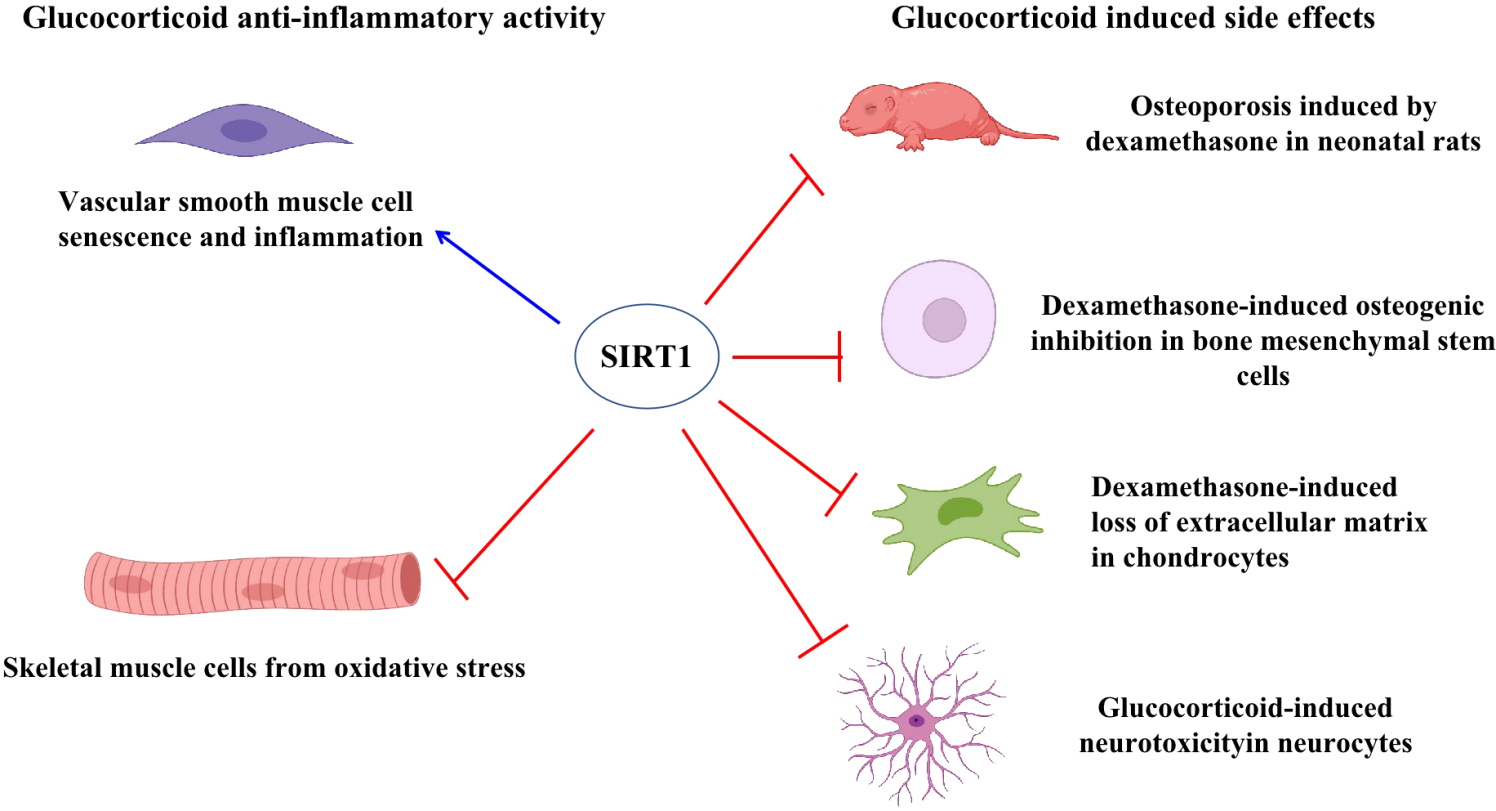
SIRT1 regulated glucocorticoid activity. SIRT1 induced glucocorticoid activity to suppress vascular smooth muscle cell senescence and inflammation. Glucocorticoids induced UPC3 expression to protect skeletal muscle cells from oxidative stress while SIRT1 inhibited UPC3 expression. SIRT1 suppressed glucocorticoid-induced side effects in different cells and rats, including reduced osteoporosis in neonatal rats, decreased loss of extracellular matrix in chondrocytes, and reduced neurotoxicity in neurocytes.

Side effects associated with glucocorticoid treatment include osteoporosis, decreased bone mineral content, and reduced bone tissue absorption. Recent study has shown that ferulic acid can protect against dexamethasone-induced osteoporosis in neonatal rats by up-regulating SIRT1 gene and protein expression and reducing nuclear factor-κB (NF-κB) activation (33). Furthermore, nicotinamide mononucleotide treatment has been shown to attenuate dexamethasone-induced osteogenic inhibition by promoting SIRT1 and peroxisome proliferator activated receptor gamma coactivator (PGC)-1α expression, while knockdown of SIRT1 reverses the protective effects of nicotinamide mononucleotide and the expression of PGC-1α (34). Dexamethasone can also induce extracellular matrix loss in chondrocytes isolated from mouse knee joints, while melatonin pretreatment reverses the negative effects of dexamethasone via mediation of the SIRT1 pathway and inhibition of SIRT1 by the inhibitor EX527 reverses the protective effects of melatonin (35). In a cellular model of corticosterone-induced neurotoxicity, d-limonene shows neuroprotective effects through up-regulation of SIRT1, thereby suppressing NF-κB nuclear translocation and inhibiting inflammatory factors (36). Thus, these results indicate that SIRT1 can suppress glucocorticoid-induced side effects (**Figure 3**).

### 4.3 SIRT1 interacts with GR

Unliganded GR is primarily localized in the cytoplasm in complex form, where it binds with heat shock proteins, immunophilins, and other molecular chaperones. Aberrant GR expression or nuclear translocation is associated with glucocorticoid insensitivity. For example, airway smooth muscle cells from patients with severe asthma exhibit reduced GR expression and impaired nuclear translocation associated with reduced glucocorticoid sensitivity (4). We previously found that decreased GR expression and impaired nuclear translocation in respiratory syncytial virus-infected cells and mice led to glucocorticoid insensitivity (37, 38). Notably, respiratory syncytial virus nonstructural protein 1 (NS1) competitively inhibits GR binding to nucleocytoplasmic transporter 13, resulting in GR cytoplasmic retention (37).

SIRT1 can influence the transcriptional activity of AR (39) as well as enhance GR-induced transcriptional activity through physical interactions. Notably, SIRT1 cooperates with GR to bind to the glucocorticoid response element-induced glucocorticoid-responsive genes (9). Knockdown of SIRT1 influenced up to 30% of the glucocorticoid-responsive genes. SIRT1 has also been shown to interact with GR in a glucocorticoid-dependent manner in rats under estradiol benzoate withdrawal (10). Furthermore, activation of hippocampal SIRT1 has been shown to block the development of postpartum depression-related to increased GR(GRα) expression (10).Thus, SIRT1 may be a novel target for the treatment of postpartum depression. In GT1–7 cells, SIRT1 knockdown using small interfering RNAs significantly suppressed GRα expression and reduced GRα protein levels (40). In contrast, GRβ, which functions as a dominant-negative inhibitor of GRα, has been reported to be elevated in corticosteroid-resistant patients with asthma, COPD, and RSV bronchiolitis (41–43). Previous studies have demonstrated that intrauterine growth retardation significantly increases H3K9 acetylation at the GRβ exon region, leading to upregulated GRβ expression in the rat hippocampus (44). Notably, H3K9 is a known deacetylation target of SIRT1 (45).It suggested that SIRT1 might play a role in the suppression of GRβ expression. However, it is important to note that there is currently no direct evidence no direct evidence to substantiate the claim that SIRT1 suppresses GRβ expression. The relationship between them and their role in glucocorticoid resistance still warrants further investigation.

Recent study has shown that hyperacetylation of Hsp90 which activity is required for the maintenance of GR stability blocks GR nuclear translocation in INS-1 cells reversed dexamethasone effect on insulin secretion (46). Deacetylation of Hsp90 at K294 by SIRT2 overexpression results in disassociation of Hsp90 with GR and subsequent nuclear translocation (47), thereby repressing inflammatory cytokine expression. There were no reports about SIRT1 interacted with Hsp90 yet while the potential ability exist.

**Figure 4:**
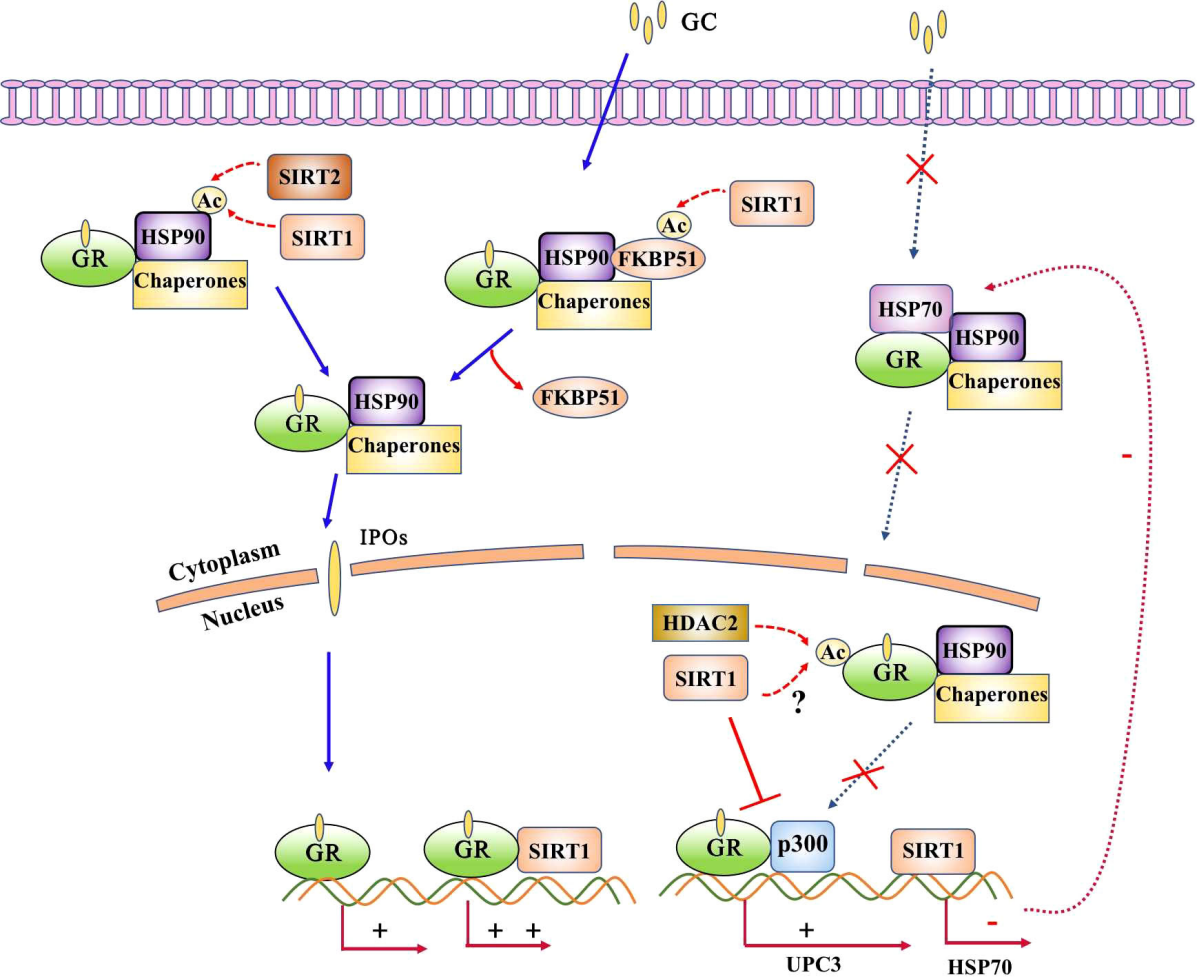
SIRT1 interacts with glucocorticoid receptor (GR). GR is predominantly localized in the cytoplasm in complex form, where it binds with heat shock proteins, immunophilins, and other molecular chaperones. Acetylation of Hsp90 results in impaired nuclear translocation, and acetylation of GR reduces its binding affinity to DNA elements as well as its ability to regulate transcription. SIRT1 directly interacts with GR and enhances it transcriptional activity and inhibits GR binding with p300. The potential ability of SIRT1 deacetylates Hsp90 and GR exsit as SIRT2 deacetylates Hsp90 or HDAC2 deacetylates GR.

Post-translational modification of GR plays an important role in regulating the biological actions of glucocorticoids. GR is acetylated by CLOCK at lysine residues 494 and 495 within the hinge region, which reduces the binding affinity of GR to DNA elements as well as its ability to regulate transcription (48). The deacetylation of GR by histone deacetylase 2 is required for efficient transrepression of NF-κB-regulated genes (49). While, SIRT1 interacted with GR whether could deacetylate these lysines residues and change the GR activity still need further investigation but the potential ability exist (**Figure 4**).

## 5 SIRT1 in glucocorticoid-resistant diseases

### 5.1 SIRT1 expression in helper T cells

**Figure 5:**
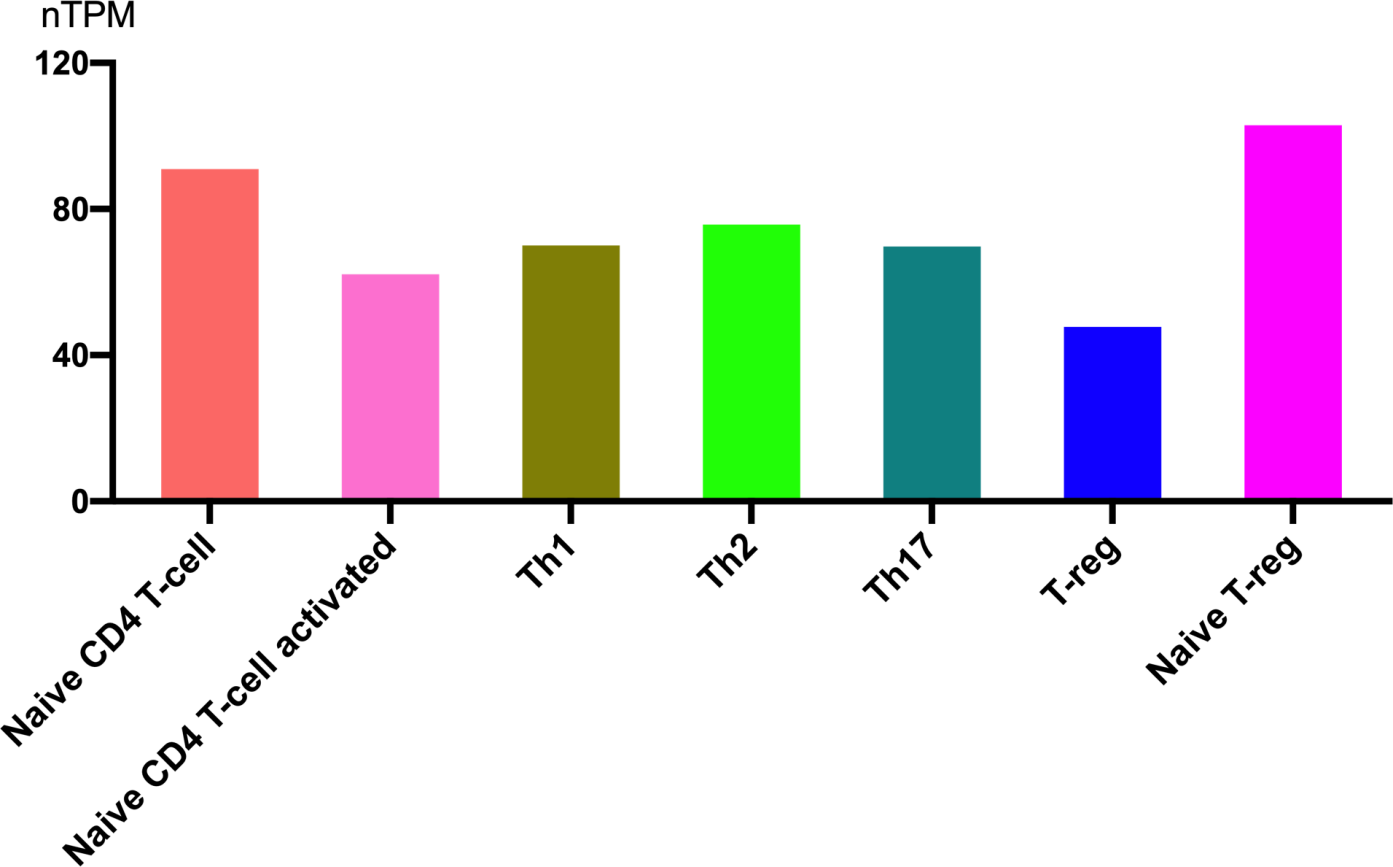
SIRT1 gene expression in T cell subsets.

Helper T cells exhibit distinct sensitivities to glucocorticoids, which significantly influence their survival, differentiation, and cytokine production. Therefore, investigating the expression and regulatory role of SIRT1 in T cell subsets is of significant importance.SIRT1 is highly expressed in immune cells, and the expression levels are nearly equivalent among helper T cell subsets. **Figure 5** shows RNA expression levels in different T cell subsets (from the Human Protein Atlas). The expression is highest in naive T-reg cells and lowest in T-reg cells. It seems that as T cells become activated, the expression of SIRT1 decreases accordingly.

### 5.2 The regulatory role of SIRT1 in T cell differentiation and glucocorticoid resistance

#### 5.2.1 Treg

Foxp3^+^ Treg cells play a crucial role in glucocorticoid-mediated suppression of eosinophilic airway inflammation, as evidenced by the failure of glucocorticoids to attenuate inflammation in Treg-depleted mice (50). In patients with nephrotic syndrome, an elevated Foxp3^+^ Tregs ratio in peripheral blood is associated with enhanced glucocorticoid sensitivity (51). Furthermore, in glucocorticoid-resistant acute Graft-versus-Host Disease (GVHD), mesenchymal stem cell treatment has been shown to increase Foxp3^+^ Tregs population, leading to significant alleviation of disease severity in murine models (52).Additionally, research showed that LINC01512 promotes SIRT1 expression in Treg cells, enhancing their differentiation and alleviating inflammation in necrotizing enterocolitis (53). However, in chronic periodontitis, SIRT1 expression is increased in CD4^+^ T cells, which suppresses Treg cells and disrupts the Th17/Treg balance, leading to persistent local inflammation (54). Studies have shown that inhibiting SIRT1 can enhance Foxp3 mRNA transcription and acetylation, thereby increasing the number and function of Treg cells (55). SIRT1 inhibitors can also enhance the function and stability of Treg cells, reducing inflammatory responses (56).

#### 5.2.2 Th17

Previous studies have demonstrated that pathogenic Th17 cells represent a distinct subset of pro-inflammatory and glucocorticoid-resistant Th17 cells in humans. These pathogenic Th17 cells are characterized by the CCR6^+^CXCR3^+^ phenotype and co-express IL-17A and IFN-γ, whereas nonpathogenic Th17 cells exhibit the CCR6^+^CCR4^+^ phenotype and produce IL-17A without IFN-γ (57). SIRT1 appears to play a regulatory role in this process, as it promotes CCR4 expression, with CCR4 levels being significantly reduced in SIRT1 knockout cells (58). This suggests that SIRT1 may preferentially enhance the development of nonpathogenic Th17 cells, potentially promoting glucocorticoid responsiveness in inflammatory diseases. Recent research has further elucidated the mechanisms underlying glucocorticoid resistance, showing that IL-1β induces STAT5-mediated glucocorticoid resistance in Th17 cells, which suppresses glucocorticoid-induced anti-inflammatory genes in experimental autoimmune encephalomyelitis (EAE) mice. Importantly, Th17-specific deletion of STAT5 abolished the IL-1β-induced glucocorticoid resistance, rendering EAE mice sensitive to glucocorticoid treatment (59). SIRT1 has been demonstrated to interact with STAT5 through direct binding, leading to STAT5 deacetylation and substantial suppression of STAT5 phosphorylation, which subsequently alleviates growth hormone resistance in mice (60).Consistent with these findings, another study revealed that active SIRT1 downregulates STAT5 expression and suppresses pSTAT5 signaling (61). Furthermore, SIRT1 has also been shown to deacetylate STAT3, inhibiting its nuclear translocation and binding to the ROR-γt promoter, thereby suppressing Th17 cell differentiation (62). This mechanism is particularly relevant given that increased p-STAT3 expression in Th17 cells has been associated with glucocorticoid insensitivity in a neutrophilic airway inflammation mouse model (63). Collectively, these findings suggest that SIRT1 may restore glucocorticoid sensitivity in Th17 cells by targeting either STAT5 or STAT3 signaling pathways. Conversely, one study showed in inflammatory bowel disease, SIRT1 expression is upregulated, and specific inhibition of SIRT1 significantly reduces ROR-γt mRNA levels in Th17 cells, leading to a decrease in Th17 cell proportion and alleviation of local inflammation (64).

#### 5.2.3 Th2

In allergic inflammation models, SIRT1 inhibits Th2 cell differentiation by suppressing the mTORC2-IL-4-STAT6-GATA3 signaling axis (65). However, in allergic rhinitis models, SIRT1 expression is increased in CD4^+^ T cells, where it promotes Th2 cell proliferation by downregulating FasL, caspase-3, and p53 expression (66).

#### 5.2.4 Th1

In Th1 cells, SIRT1 expression levels are relatively high, but Foxo1 retention in the nucleus is limited. Treatment with Ex527 did not significantly alter IFN-γ secretion (67). In bronchiolitis obliterans following lung transplantation, SIRT1 expression is reduced in peripheral blood T cells, leading to diminished responsiveness to glucocorticoids. Activation of SIRT1 enhances the inhibitory effect of glucocorticoids on IFN-γ and TNF-α production in T cells (68).

Given the diverse functions of SIRT1 across different T cell subsets and inflammatory microenvironments, its role in modulating glucocorticoid responses may remain complex. Further direct evidence are needed to elucidate how SIRT1 regulates glucocorticoid responses in T cells.

### 5.3 The role of SIRT1 in other glucocorticoid-resistant diseases

Accumulating evidence highlights the important role of SIRT1 in glucocorticoid-resistant diseases. SIRT1 single-nucleotide polymorphisms are associated with glucocorticoid sensitivity in primary immune thrombocytopenia (69). Furthermore, CC/TC genotypes of SIRT1 rs12778366 show a two-fold increase in the risk of glucocorticoid resistance (70). CD8^+^ T and natural killer T cell(NKT)-like cells in patients with chronic obstructive pulmonary disease (COPD) show glucocorticoid resistance associated with decreased SIRT1 expression (71). Treatment with SIRT1 activators restores the anti-inflammatory activity of prednisolone and reduces pro-inflammatory cytokine production (72). Glucocorticoids are also widely used to treat B acute lymphoblastic leukemia, although their efficacy is often impaired by the development of resistance (73). FOXO3a translocates into the nucleus to mediate the cytotoxic function of dexamethasone, and SIRT1/2-mediated acetylation of Lys-242/5 is associated with dexamethasone-induced FOXO3a activity (73). Human peripheral blood mononuclear cells (PBMCs) from patients with severe asthma show reduced SIRT1 protein expression and activity and increased Th2 cytokine expression (74); and treatment of HUT78 T-cells with SIRT inhibitors can increase GATA Binding Protein 3(GATA-3) acetylation and IL-4 and IL-13 expression (75). However, severe asthmatics appear to be largely unresponsive to high-dose inhaled and systemic glucocorticoids (76). While direct evidence showing an association between SIRT1 and glucocorticoid resistance in asthma is still lacking, studies have demonstrated that SIRT1 plays a critical role in suppressing allergic airway inflammation *in vivo* and *in vitro* (77).

## 6 Concluding remarks and future perspectives

Glucocorticoid resistance and reduced sensitivity are unresolved issues in severe asthma and other diseases. SIRT1 can regulate glucocorticoid activity and interact with GR. Thus, Activation or inhibition of SIRT1 represents a promising novel therapeutic strategy for clinical trials and therapeutic applications. Further studies are still needed to determine the role of SIRT1 and glucocorticoids and the therapeutic activity of SIRT1 in glucocorticoid-resistant diseases.

## References

[1] Enweasor C Flayer CH Haczku A. Ozone-induced oxidative stress, neutrophilic airway inflammation, and glucocorticoid resistance in asthma. Front Immunol. (2021) 12:631092. doi: 10.3389/fimmu.2021.631092 33717165 PMC7952990

[2] Trautmann A Vivarelli M Samuel S International Pediatric Nephrology Association. IPNA clinical practice recommendations for the diagnosis and management of children with steroid-resistant nephrotic syndrome. Pediatr Nephrol. (2020) 35:1529–61. doi: 10.1007/s00467-020-04519-1 PMC7316686 32382828

[3] Ishida A Ohta N Koike S Aoyagi M Yamakawa M. Overexpression of glucocorticoid receptor-beta in severe allergic rhinitis. Auris Nasus Larynx. (2010) 37:584–8. doi: 10.1016/j.anl.2009.12.002 20185258

[4] Chang PJ Michaeloudes C Zhu J Shaikh N Baker J Chung KF. Impaired nuclear translocation of the glucocorticoid receptor in corticosteroid-insensitive airway smooth muscle in severe asthma. Am J Respir Crit Care Med. (2015) 191:54–62. doi: 10.1164/rccm.201402-0314OC 25411910 PMC4299627

[5] Mei D Tan WSD Wong WSF. Pharmacological strategies to regain steroid sensitivity in severe asthma and COPD. Curr Opin Pharmacol. (2019) 46:73–81. doi: 10.1016/j.coph.2019.04.010 31078066

[6] Hentati-Kallel M Le Jan S Bernard P Antonicelli F Trussardi-Régnier A. Histone deacetylases meet microRNA-associated MMP-9 expression regulation in glucocorticoid-sensitive and -resistant cell lines. Int J Oncol. (2017) 50:717–26. doi: 10.3892/ijo.2016.3830 28035377

[7] Tsapis M Lieb M Manzo F Shankaranarayanan P Herbrecht R Lutz P. HDAC inhibitors induce apoptosis in glucocorticoid-resistant acute lymphatic leukemia cells despite a switch from the extrinsic to the intrinsic death pathway. Int J Biochem Cell Biol. (2007) 39:1500–9. doi: 10.1016/j.biocel.2007.03.009 17499001

[8] Mishra R Chaturvedi R Hashim Z Nath A Khan A Gupta M. Role of P-gp and HDAC2 and their reciprocal relationship in uncontrolled asthma. Curr Pharm Biotechnol. (2021) 22:408–13. doi: 10.2174/1389201021666200529104042 32469696

[9] Suzuki S Iben JR Coon SL Kino T. SIRT1 is a transcriptional enhancer of the glucocorticoid receptor acting independently to its deacetylase activity. Mol Cell Endocrinol. (2018) 461:178–87. doi: 10.1016/j.mce.2017.09.012 PMC5756502 28923345

[10] Wang J Ma SF Yun Q Liu WJ Guo MN Zhu YQ. Ameliorative effect of SIRT1 in postpartum depression mediated by upregulation of the glucocorticoid receptor. Neurosci Lett. (2021) 761:136112. doi: 10.1016/j.neulet.2021.136112 34265417

[11] Rajabi N Galleano I Madsen AS Olsen CA. Targeting sirtuins: substrate specificity and inhibitor design. Prog Mol Biol Transl Sci. (2018) 154:25–69. doi: 10.1016/bs.pmbts.2017.11.003 29413177

[12] Shin MK Vázquez-Rosa E Koh Y Dhar M Chaubey K Cintrón-Pérez CJ. Reducing acetylated tau is neuroprotective in brain injury. Cell. (2021) 184:2715–32.e23. doi: 10.1016/j.cell.2021.03.032 33852912 PMC8491234

[13] Katsyuba E Mottis A Zietak M De Franco F van der Velpen V Gariani K. *De novo* NAD+ synthesis enhances mitochondrial function and improves health. Nature. (2018) 563:354–9. doi: 10.1038/s41586-018-0645-6 PMC6448761 30356218

[14] Kumar A Chauhan S. How much successful are the medicinal chemists in modulation of SIRT1: A critical review. Eur J Med Chem. (2016) 119:45–69. doi: 10.1016/j.ejmech.2016.04.063 27153347

[15] Kang H Suh JY Jung YS Kim MK Chung JH. Peptide switch is essential for Sirt1 deacetylase activity. Mol Cell. (2011) 44:203–13. doi: 10.1016/j.molcel.2011.07.038 PMC3240942 22017869

[16] Tanno M Sakamoto J Miura T Shimamoto K Horio Y. Nucleocytoplasmic shuttling of the NAD+-dependent histone deacetylase SIRT1. J Biol Chem. (2007) 282:6823–32. doi: 10.1074/jbc.M609554200 17197703

[17] Vaquero A Scher M Lee D Erdjument-Bromage H Tempst P Reinberg D. Human SirT1 interacts with histone H1 and promotes formation of facultative heterochromatin. Mol Cell. (2004) 16:93–105. doi: 10.1016/j.molcel.2004.08.031 15469825

[18] Nakahata Y Kaluzova M Grimaldi B Sahar S Hirayama J Chen D. The NAD+-dependent deacetylase SIRT1 modulates CLOCK-mediated chromatin remodeling and circadian control. Cell. (2008) 134:329–40. doi: 10.1016/j.cell.2008.07.002 PMC3526943 18662547

[19] Park JH Lee SW Yang SW Yoo HM Park JM Seong MW. Modification of DBC1 by SUMO2/3 is crucial for p53-mediated apoptosis in response to DNA damage. Nat Commun. (2014) 5:5483. doi: 10.1038/ncomms6483 25406032

[20] Wahab F Rodriguez Polo I Behr R. SIRT1 expression and regulation in the primate testis. Int J Mol Sci. (2021) 22:3207. doi: 10.3390/ijms22063207 33809872 PMC8004242

[21] Langley E Pearson M Faretta M Bauer UM Frye RA Minucci S. Human SIR2 deacetylates p53 and antagonizes PML/p53-induced cellular senescence. EMBO J. (2002) 21:2383–96. doi: 10.1093/emboj/21.10.2383 PMC126010 12006491

[22] Yamamori T DeRicco J Naqvi A Hoffman TA Mattagajasingh I Kasuno K. SIRT1 deacetylates APE1 and regulates cellular base excision repair. Nucleic Acids Res. (2010) 38:832–45. doi: 10.1093/nar/gkp1039 PMC2817463 19934257

[23] Yu H Kim YM Cho M. Cytoplasm-localized SIRT1 downregulation attenuates apoptosis and cell cycle arrest in cisplatin-resistant lung cancer A549 cells. J Cancer. (2020) 11:4495–509. doi: 10.7150/jca.44383 PMC7255359 32489467

[24] Yanagisawa S Baker JR Vuppusetty C Vuppusetty C Koga T Colley T. The dynamic shuttling of SIRT1 between cytoplasm and nuclei in bronchial epithelial cells by single and repeated cigarette smoke exposure. PLoS One. (2018) 13:e0193921. doi: 10.1371/journal.pone.0193921 29509781 PMC5839577

[25] Merlo S Luaces JP Spampinato SF Toro-Urrego N Caruso GI D'Amico F. SIRT1 mediates melatonin's effects on microglial activation in hypoxia: *in vitro* and *in vivo* evidence. Biomolecules. (2020) 10:364. doi: 10.3390/biom10030364 32120833 PMC7175216

[26] Pan S Cui Y Fu Z Zhang L Xing H. MicroRNA-128 is involved in dexamethasone-induced lipid accumulation via repressing SIRT1 expression in cultured pig preadipocytes. J Steroid Biochem Mol Biol. (2019) 186:185–95. doi: 10.1016/j.jsbmb.2018.10.013 30394333

[27] Murray MY Rushworth SA Zaitseva L Bowles KM Macewan DJ. Attenuation of dexamethasone-induced cell death in multiple myeloma is mediated by miR-125b expression. Cell Cycle. (2013) 12:2144–53. doi: 10.4161/cc.25251 PMC3737316 23759586

[28] Lee H Kim M Park YH Park JB. Dexamethasone downregulates SIRT1 and IL6 and upregulates EDN1 genes in stem cells derived from gingivae via the AGE/RAGE pathway. Biotechnol Lett. (2018) 40:509–19. doi: 10.1007/s10529-017-2493-0 29302812

[29] Pasquereau S Totoson P Nehme Z Abbas W Kumar A Verhoeven F. Impact of glucocorticoids on systemic sirtuin 1 expression and activity in rats with adjuvant-induced arthritis. Epigenetics. (2021) 16:132–43. doi: 10.1080/15592294.2020.1790789 PMC7889198 32615849

[30] Moore RL Dai Y Faller DV. Sirtuin 1 (SIRT1) and steroid hormone receptor activity in cancer. J Endocrinol. (2012) 213:37–48. doi: 10.1530/JOE-11-0217 22159506 PMC3804056

[31] Sung JY Kim SG Kim JR Choi HC. Prednisolone suppresses adriamycin-induced vascular smooth muscle cell senescence and inflammatory response via the SIRT1-AMPK signaling pathway. PLoS One. (2020) 15:e0239976. doi: 10.1371/journal.pone.0239976 32997729 PMC7526920

[32] Amat R Solanes G Giralt M Villarroya F. SIRT1 is involved in glucocorticoid-mediated control of uncoupling protein-3 gene transcription. J Biol Chem. (2007) 282:34066–76. doi: 10.1074/jbc.M707114200 17884810

[33] Hou T Zhang L Yang X. Ferulic acid, a natural polyphenol, protects against osteoporosis by activating SIRT1 and NF-κB in neonatal rats with glucocorticoid-induced osteoporosis. BioMed Pharmacother. (2019) 120:109205. doi: 10.1016/j.biopha.2019.109205 31634777

[34] Huang RX Tao J. Nicotinamide mononucleotide attenuates glucocorticoid−induced osteogenic inhibition by regulating the SIRT1/PGC−1α signaling pathway. Mol Med Rep. (2020) 22:145–54. doi: 10.3892/mmr.2020.11116 PMC7248519 32377728

[35] Yang W Kang X Qin N Li F Jin X Ma Z. Melatonin protects chondrocytes from impairment induced by glucocorticoids via NAD+-dependent SIRT1. Steroids. (2017) 126:24–9. doi: 10.1016/j.steroids.2017.08.005 28803211

[36] Tang XP Guo XH Geng D Weng LJ. d-Limonene protects PC12 cells against corticosterone-induced neurotoxicity by activating the AMPK pathway. Environ Toxicol Pharmacol. (2019) 70:103192. doi: 10.1016/j.etap.2019.05.001 31103492

[37] Xie J Long X Gao L Chen S Zhao K Li W. Respiratory syncytial virus nonstructural protein 1 blocks glucocorticoid receptor nuclear translocation by targeting IPO13 and may account for glucocorticoid insensitivity. J Infect Dis. (2017) 217:35–46. doi: 10.1093/infdis/jix445 28968829

[38] Gao L Mo S Xie J Chen S Wen X Long X. Respiratory syncytial virus nonstructural protein 1 downregulates glucocorticoid receptor expression through miR-29a. J Allergy Clin Immunol. (2019) 144:854–57.e6. doi: 10.1016/j.jaci.2019.05.014 31128120

[39] Montie HL Pestell RG Merry DE. SIRT1 modulates aggregation and toxicity through deacetylation of the androgen receptor in cell models of SBMA. J Neurosci. (2011) 31:17425–36. doi: 10.1523/JNEUROSCI.3958-11.2011 PMC6088793 22131404

[40] Yang G Wan L Zhang S Shi X Wang J Hu L. CLOCK, SIRT1, and HDAC2 knockdown along with melatonin intervention significantly decreased the level glucocorticoid receptor. RUSS J Genet. (2022) 58:85–93. doi: 10.1134/s1022795422010148

[41] Diaz PV Pinto RA Mamani R Uasapud PA Bono MR Gaggero AA. Increased expression of the glucocorticoid receptor β in infants with RSV bronchiolitis. Pediatrics. (2012) 130:e804–11. doi: 10.1542/peds.2012-0160 23008453

[42] Butler CA McQuaid S Taggart CC Weldon S Carter R Skibinski G. Glucocorticoid receptor β and histone deacetylase 1 and 2 expression in the airways of severe asthma. Thorax. (2012) 67:392–8. doi: 10.1136/thoraxjnl-2011-200760 22156779

[43] Milara J Lluch J Almudever P Freire J Xiaozhong Q Cortijo J. Roflumilast N-oxide reverses corticosteroid resistance in neutrophils from patients with chronic obstructive pulmonary disease. J Allergy Clin Immunol. (2014) 134:314–22. doi: 10.1016/j.jaci.2014.02.001 24636089

[44] Ke X Schober ME McKnight RA O'Grady S Caprau D Yu X. Intrauterine growth retardation affects expression and epigenetic characteristics of the rat hippocampal glucocorticoid receptor gene. Physiol Genomics. (2010) 42:177–89. doi: 10.1152/physiolgenomics.00201.2009 20388836

[45] Yang Y Peng W Su X Yue B Shu S Wang J. Epigenomics analysis of the suppression role of SIRT1 via H3K9 deacetylation in preadipocyte differentiation. Int J Mol Sci. (2023) 24:11281. doi: 10.3390/ijms241411281 37511041 PMC10379189

[46] Zhu K Zhang Y Zhang J Zhou F Zhang L Wang S. Acetylation of Hsp90 reverses dexamethasone-mediated inhibition of insulin secretion. Toxicol Lett. (2020) 320:19–27. doi: 10.1016/j.toxlet.2019.11.022 31778773

[47] Sun K Wang X Fang N Xu A Lin Y Zhao X. SIRT2 suppresses expression of inflammatory factors via Hsp90-glucocorticoid receptor signalling. J Cell Mol Med. (2020) 24:7439–50. doi: 10.1111/jcmm.15365 PMC7339210 32515550

[48] Nader N Chrousos GP Kino T. Circadian rhythm transcription factor CLOCK regulates the transcriptional activity of the glucocorticoid receptor by acetylating its hinge region lysine cluster: potential physiological implications. FASEB J. (2009) 23:1572–83. doi: 10.1096/fj.08-117697 PMC2669420 19141540

[49] Ito K Yamamura S Essilfie-Quaye S Cosio B Ito M Barnes PJ. Histone deacetylase 2-mediated deacetylation of the glucocorticoid receptor enables NF-kappaB suppression. J Exp Med. (2006) 203:7–13. doi: 10.1084/jem.20050466 16380507 PMC2118081

[50] Nguyen QT Kim D Iamsawat S Le HT Kim S Qiu KT. Cutting edge: steroid responsiveness in foxp3+ Regulatory T cells determines steroid sensitivity during allergic airway inflammation in mice. J Immunol. (2021) 207:765–70. doi: 10.4049/jimmunol.2100251 PMC8323959 34301840

[51] Jaiswal A Prasad N Agarwal V Yadav B Tripathy D Rai M. Regulatory and effector T cells changes in remission and resistant state of childhood nephrotic syndrome. Indian J Nephrol. (2014) 24:349–55. doi: 10.4103/0971-4065.132992 PMC4244713 25484527

[52] Wu R Liu C Deng X Chen L Hao S Ma L. Enhanced alleviation of aGVHD by TGF-β1-modified mesenchymal stem cells in mice through shifting MΦ into M2 phenotype and promoting the differentiation of Treg cells. J Cell Mol Med. (2020) 24:1684–99. doi: 10.1111/jcmm.14862 PMC6991663 31782262

[53] Gao X Liu W Gao P Li S Chen Z Ma F. Melatonin-induced lncRNA LINC01512 prevents Treg/Th17 imbalance by promoting SIRT1 expression in necrotizing enterocolitis. Int Immunopharmacol. (2021) 96:107787. doi: 10.1016/j.intimp.2021 34162151

[54] Zheng Y Dong C Yang J Jin Y Zheng W Zhou Q. Exosomal microRNA-155-5p from PDLSCs regulated Th17/Treg balance by targeting sirtuin-1 in chronic periodontitis. J Cell Physiol. (2019) 234:20662–74. doi: 10.1002/jcp.28671 31016751

[55] Chadha S Wang L Hancock WW Beier UH. Sirtuin-1 in immunotherapy: A Janus-headed target. J Leukoc Biol. (2019) 106:337–43. doi: 10.1002/JLB.2RU1118-422R PMC7477756 30605226

[56] Jadon N Shanthalingam S Tew GN Minter LM. PRMT5 regulates epigenetic changes in suppressive Th1-like iTregs in response to IL-12 treatment. Front Immunol. (2024) 14:1292049. doi: 10.3389/fimmu.2023.1292049 38259494 PMC10800960

[57] Ramesh R Kozhaya L McKevitt K Djuretic IM Carlson TJ Quintero MA. Pro-inflammatory human Th17 cells selectively express P-glycoprotein and are refractory to glucocorticoids. J Exp Med. (2014) 211:89–104. doi: 10.1084/jem.20130301 24395888 PMC3892977

[58] Fang Y Fan W Xu X Janoshazi AK Fargo DC Li X. SIRT1 regulates cardiomyocyte alignment during maturation. J Cell Sci. (2022) 135:jcs259076. doi: 10.1242/jcs.259076 35260907 PMC9016619

[59] Miller-Little WA Chen X Salazar V Liu C Bulek K Zhou JY. A TH17-intrinsic IL-1β-STAT5 axis drives steroid resistance in autoimmune neuroinflammation. Sci Immunol. (2024) 9:eabq1558. doi: 10.1126/sciimmunol.abq1558 38701190

[60] Yamamoto M Iguchi G Fukuoka H Suda K Bando H Takahashi M. SIRT1 regulates adaptive response of the growth hormone–insulin-like growth factor-I axis under fasting conditions in liver. Proc Natl Acad Sci U.S.A. (2013) 110:14948–53. doi: 10.1073/pnas PMC3773795 23980167

[61] Gardner PJ Joshi L Lee RW Dick AD Adamson P Calder VL. SIRT1 activation protects against autoimmune T cell-driven retinal disease in mice via inhibition of IL-2/Stat5 signaling. J Autoimmun. (2013) 42:117–29. doi: 10.1016/j.jaut.2013.01.011 23395551

[62] Limagne E Thibaudin M Euvrard R Berger H Chalons P Végan F. Sirtuin-1 activation controls tumor growth by impeding th17 differentiation via STAT3 deacetylation. Cell Rep. (2017) 19:746–59. doi: 10.1016/j.celrep.2017.04.004 28445726

[63] Fang SB Zhang HY Jiang AY Fan XL Lin YD Li CL. Human iPSC-MSCs prevent steroid-resistant neutrophilic airway inflammation via modulating Th17 phenotypes. Stem Cell Res Ther. (2018) 9:147. doi: 10.1186/s13287-018-0897-y 29793557 PMC5968555

[64] Zhu Y Wang Y Zuo X Liu S Cao L Wang J. Inhibition SIRT1 to regulate FOXP3 or RORγt can restore the balance of Treg/Th17 axis in ulcerative colitis and enhance the anti-inflammatory effect of moxibustion. Front Immunol. (2025) 15:1525469. doi: 10.3389/fimmu.2024.1525469 39867884 PMC11757129

[65] Pandit M Timilshina M Gu Y Acharya S Chung Y Seo SU. AMPK suppresses Th2 cell responses by repressing mTORC2. Exp Mol Med. (2022) 54:1214–24. doi: 10.1038/s12276-022-00832-x PMC9440126 35999454

[66] Hu T Fan X Ma L Liu J Chang Y Yang P. TIM4-TIM1 interaction modulates Th2 pattern inflammation through enhancing SIRT1 expression. Int J Mol Med. (2017) 40:1504–10. doi: 10.3892/ijmm.2017.3150 PMC5627870 28949386

[67] Chatterjee S Daenthanasanmak A Chakraborty P Wyatt MW Dhar P Selvam SP. CD38-NAD+Axis regulates immunotherapeutic anti-tumor T cell response. Cell Metab. (2018) 27:85–100.e8. doi: 10.1016/j.cmet.2017.10.006 29129787 PMC5837048

[68] Hodge G Hodge S Liu H Nguyen P Holmes-Liew CL Holmes M. BOS is associated with decreased SIRT1 in peripheral blood proinflammatory T, NK, and NKT-like lymphocytes. Transplantation. (2019) 103:2255–63. doi: 10.1097/TP.0000000000002817 31651733

[69] Wang S Zhang X Leng S Zhang Y Li J Peng J. SIRT1 single-nucleotide polymorphisms are associated with corticosteroid sensitivity in primary immune thrombocytopenia patients. Ann Hematol. (2021) 100:2453–62. doi: 10.1007/s00277-021-04583-z 34269838

[70] Hodge G Tran HB Reynolds PN Jersmann H Hodge S. Lymphocyte senescence in COPD is associated with decreased sirtuin 1 expression in steroid resistant pro-inflammatory lymphocytes. Ther Adv Respir Dis. (2020) 14:1753466620905280. doi: 10.1177/1753466620905280 32270742 PMC7153179

[71] Consolaro F Ghaem-Maghami S Bortolozzi R Zona S Khongkow M Basso G. FOXO3a and posttranslational modifications mediate glucocorticoid sensitivity in B-ALL. Mol Cancer Res. (2015) 13:1578–90. doi: 10.1158/1541-7786.MCR-15-0127 26376801

[72] Zhang YZ Wu QJ Yang X Xing XX Chen YY Wang H. Effects of SIRT1/Akt pathway on chronic inflammatory response and lung function in patients with asthma. Eur Rev Med Pharmacol Sci. (2019) 23:4948–53. doi: 10.26355/eurrev_201906_18085 31210330

[73] Colley T Mercado N Kunori Y Brightling C Bhavsar PK Barnes PJ. Defective sirtuin-1 increases IL-4 expression through acetylation of GATA-3 in patients with severe asthma. J Allergy Clin Immunol. (2016) 137:1595–97.e7. doi: 10.1016/j.jaci.2015.10.013 26627546

[74] Israel E Reddel HK. Severe and difficult-to-treat asthma in adults. N Engl J Med. (2017) 377:965–76. doi: 10.1056/NEJMra1608969 28877019

[75] Lai T Su G Wu D Chen Z Chen Y Yi H. Myeloid-specific SIRT1 deletion exacerbates airway inflammatory response in a mouse model of allergic asthma. Aging (Albany NY). (2021) 13:15479–90. doi: 10.18632/aging.203104 PMC8221322 34099590

